# Predictive, preventive and personalized medicine (PPPM) as a strategic avenue and global tool for advancing T1D-related care: *Fundamental*, *Applied and Affiliated Issues*

**DOI:** 10.1186/1878-5085-5-S1-A69

**Published:** 2014-02-11

**Authors:** Sergey Suchkov, Olga Golubnitschaja, Matt von Herrath, Paolo Pozzilli, Mihail Paltsev, Ashot Mkrtumyan, Martin Frank, Trevor Marshall, Harry Schroeder

**Affiliations:** 1 I.M.Sechenov First Moscow State Medical University, Moscow, Russia; 2 A.I.Evdokimov Moscow State Medical & Dental University, Moscow, Russia; 3 Rheinische Friedrich-Wilhelms-University of Bonn, Bonn, Germany; 4 Department of Endocrinology and Diabetes, Rome University Campus Bio-Medico, Rome, Italy; 5 National Research Center “Kurchatov’s Institute”, Moscow, Russia; 6 Type 1 Diabetes Research Center, La Jolla Institute for Allergy and Immunology, UCSF in La Jolla, La Jolla, CA, USA; 7 Type 1 Diabetes R&D Center, Seattle, USA; 8 Center for Autoimmune Diseases and Autoimmune Foundation, LA, CA, USA; 9 Centre for Biophotonics LEK, Lancaster University, Lancaster, UK; 10 Departments of Medicine, Genetics, Comparative Pathology, Laboratory and Microbiology, University of Alabama at Birmingham, Birmingham, AL 35294, USA

## 

Type one diabetes (T1D: (juvenile onset) whilst being a chronic disease, the defining feature of which is the destruction of the insulin-secreting *beta*-cells and subsequent dependence on exogenous insulin, is possessing with hallmark characteristics of the complex T cell-mediated autoimmunity superimposed on genetic susceptibility. Both genetic and environmental factors combine to precipitate disease, and the outcome of the pathological process is dependent on multiple interrelated factors. The annual global incidence of T1D is increasing by 3-5% per year.

Both HLA-genes and immune markers (autoantibodies/autoAbs) have been validated as *predictive* biomarkers of the subsequent development of the disease in higher-risk relatives and the lower-risk general population. Over the last three decades, using a combination of genomic, proteomic, and metabolomic biomarkers, clinicians are now able to quantify an individual's disease risk from 1 in 100,000 to more than 1 in 2. The HLA genes are reported to account for approximately 40-50% of the familial aggregation of T1D.

Current approaches for the *prediction* of T1D in screening studies take advantage of genotyping HLA-DR and HLA-DQ loci, which is then combined with family history and screening for autoAbs directed against islet-cell Ags. Inclusion of additional moderate HLA risk haplotypes may help identify the majority of children withT1D before the onset of the disease. Fine mapping and functional studies are gradually revealing the complex mechanisms whereby immune self-tolerance is lost, involving adaptive immunity. Genetic *prediction* of T1D risks is showing promise of use for *preventive* strategies. And understanding of the genetics, environmental factors, and natural history of T1D has resulted in greater understanding of the etiology and epidemiology of the disease.

The clinical care of patients with T1D has greatly improved over the past few decades; however, it remains impossible to completely normalize blood sugar utilizing currently available tools. Meanwhile, *subclinical* and *predictive* diagnostic armamentarium is still far from being unique, and innovative algorithms that significantly differ from the clinical approaches should become the major bricks to be laid into the basis of a *subclinical* diagnosis of pre-T1D.

Generally, there are three levels desirable for an optimal pre-T1D and T1D-related healthcare, namely,

*(i) 1^st^ level: prediction* of the familial predisposition, targeted *prevention* of pre-T1D early in childhood;

*(ii) 2^nd^ level: prediction* of early/premature aging and targeted *prevention* of T1D-related pre-stages;

*(iii) 3^rd^ level: prediction* of secondary complications in the cohort of T1D-patients and creation of personalized treatment modes tailored to the patient to *prevent* post-T1D complications frequently developed.

Accurate *prediction* is vital for T1D *prevention* so that *preventive* treatment can be given to those individuals who are most likely to develop the disease. *Predictive* medicine is intended more so for healthy individuals, its purpose being to determine whether susceptibility to a particular disease is increased or not. And the long asymptomatic period before the onset of clinical T1D offers good opportunities for disease *prevention*. And *preventive* portion of PPPM (or preventive care) refers to measures taken to prevent diseases rather than curing them or treating their symptoms. Indeed, many chronic diseases like T1D may be *preventable* by avoiding those factors that trigger the disease process (*primary* prevention) or by use of treatment modes that modulate the disease process before the onset of clinical symptoms (*secondary* prevention).

The utility of *predictive* biomarkers to monitor T1D-related stages and to *predict* the transitions is dependent on three key parameters, which must be carefully assessed: *sensitivity*, *specificity* and positive *predictive* value. Recent advances in genomics, proteomics, and bioinformatics offer a great opportunity for biomarker discovery, and the newly identified *biomarkers* and *biopredictors* have the potential to bring increased accuracy in T1D diagnosis and classification, as well as *personalized* therapeutic monitoring. The latter including efficacious quenching of autoimmune insulitis (pre-T1D) and *prevention* of clinical T1D will require detection of the earliest events in the process. But currently, there is no effective treatment strategy to restore glucose regulation in T1D patients. So, *personalized* medicine is a medical model that proposes the customization of healthcare, with all decisions and practices being tailored to the individual T1D patient by use of genetic or other information.

So far, antiislet autoAbs are most widely used as serum biomarker in making and verifying the diagnosis of *subclinical* and *clinical* stages of T1D, but T-cell readouts and metabolome studies might strengthen and bring forward diagnosis. The autoAbs can be detected before (as early as 5-10 years before the clinical onset) and at time of clinical diagnosis of disease and could thus be used to *predict* risks of developing T1D in first degree relatives of probands.

Monotherapy for T1D has not resulted in sustained tolerance and preservation of *C*-peptide production; individuals still progress to a complete loss of endogenous insulin producing ability. Combination therapy is likely to be necessary as different pathways and arms of the immune system can be targeted. However, current *preventive* clinical trials mostly focus on environmental triggers. At the same time, there is an immediate need to restore both *beta*-cell function and immune tolerance to control disease progression and ultimately cure T1D, and so, the strategy of *prevention* of pre-T1D-T1D transition should contain two critical points, i.e., *(i)* obligatory arrest/blockage of autoagression and *(ii)* restoration of *beta*-cell infrastructure and function. As it is evident, strategies for disease intervention, therefore, will not only require the induction of T-cell tolerance by tipping the balance towards regulation but will also need to contain approaches that result in the scavenging of inflammatory mediators, in order to facilitate repair.

For instance, efficiently halting immune attack in the islet milieu by an effector-specific manner apparently provides the *preventive* and *therapeutic* strategies in T1D. For instance, FoxP3-expressing CD4^+^ Tregs are potential candidates to control autoimmunity because they play a central role in maintaining self-tolerance.

Preservation and/or restoration of structural architectonics and function of *beta* cells can be accomplished by the use of *(i)* immune, cell-based and/or gene therapy (e.g., islet transplantation could be an appropriate treatment to accomplish insulin independence and long-term homeostasis of glucose in T1D), and *(ii)* regenerative drug-based treatment including stem cell (SC) technologies. Tremendous progress has been made in the field of islet regeneration in the past few years, and *beta*-cell replacement by islet transplantation and/or SCs has provided new hope for a cure of T1D.

However, it is difficult to protect the islet grafts or *beta*-cells restored from subsequent immune attack and prolong their survival. The optimal therapeutic approach for T1D in this connection should ideally preserve the remaining β-cells, restore β-cell function, and protect the replaced insulin-producing cells from autoimmunity. SCs possess unique immune and restorative properties that could be harnessed to improve the treatment of T1D; indeed, SCs may re-establish peripheral tolerance toward β-cells through reshaping of the immune response and inhibition of autoreactive CTCs.

Along with combination therapy, we believe targeted and specific immune therapies are needed to prevent and ultimately cure T1D. Those therapies can be designed to specifically target insulin-HLA complexes and the TCRs that recognize them. Small molecule approaches can block autoAg presentation or TCR recognition of autoAg-HLA complexes. Another strategy includes designing MAbs to recognize autoAg peptide-HLA complexes with the peptide in a defined register recognized by the autoreactive TCRs. As the structural basis of autoAg presentation and T cell recognition advances, these novel approaches become realistic. There is thus a requirement for an increased, collaborative effort between clinical endocrinologists, specialists in PPPM, stem cell biologists and immunologists in order to tailor an optimal and *personalized* therapeutic strategy for the treatment of this debilitating disease.

Moreover, implementation of PPPM program requires the adjusted technology for adequate interpretation of diagnostic data before the current model “physician-patient” could be gradually displaced by a novel model “medical advisor-healthy men-at-risk”. Systemic approach to formation of an innovative infrastructure regarding *prediction* and *prevention* algorithms is an ultimate approach that will contribute to modernization of T1D-related healthcare significantly. An understanding and possibly a complete description of the factors underlying the burden of T1D will provide policy-makers, healthcare providers and mentors with an opportunity to guide *primary* and *secondary preventive* initiatives at both individual and community levels. Our challenge is that new guidelines should create the robust juristic and economic platform for advanced medical services utilizing the cost-effective models of risk assessments followed by tailored *preventive* and *personalized* treatments focused on the precursor stages of T1D. What is a realistic timeline for the incorporation of PPPM into the practice of endocrinology and T1D, in particular? Patients and relatives alike want interventions that work right now. And PPPM would thus promise to sharply reverse the ever escalating costs of T1D-related diagnosis and treatment to stratify patients and disease, less expensive approaches to drug discovery, *preventive* medicine and wellness. PPPM also promises to improve T1D patient outcomes, and to empower both the patient and the physician.

